# Leveraging AI to Optimize Maintenance of Health Evidence and Offer a One-Stop Shop for Quality-Appraised Evidence Syntheses on the Effectiveness of Public Health Interventions: Quality Improvement Project

**DOI:** 10.2196/69700

**Published:** 2025-07-29

**Authors:** Kristin Rogers, Alanna Miller, Ashley Girgis, Emily C Clark, Sarah E Neil-Sztramko, Maureen Dobbins

**Affiliations:** 1 National Collaborating Centre for Methods and Tools, School of Nursing, McMaster University, 175 Longwood Road South, Suite 210A, Hamilton, ON, L8P0A1, Canada, 1 9055259140 ext 20450; 2 Department of Health Research Methods, Evidence, and Impact, Faculty of Health Sciences, McMaster University, Hamilton, ON, Canada; 3 School of Nursing, Faculty of Health Sciences, McMaster University, Hamilton, ON, Canada

**Keywords:** machine learning, natural language processing, automation, title and abstract screening, text classification, database management, citation screening, methodology, systematic review

## Abstract

**Background:**

Health Evidence provides access to quality appraisals for >10,000 evidence syntheses on the effectiveness and cost-effectiveness of public health and health promotion interventions. Maintaining Health Evidence has become increasingly resource-intensive due to the exponential growth of published literature. Innovative screening methods using artificial intelligence (AI) can potentially improve efficiency.

**Objective:**

The objectives of this project are to: (1) assess the ability of AI-assisted screening to correctly predict nonrelevant references at the title and abstract level and investigate the consistency of this performance over time, and (2) evaluate the impact of AI-assisted screening on the overall monthly manual screening set.

**Methods:**

Training and testing were conducted using the DistillerSR AI Preview & Rank feature. A set of manually screened references (n=43,273) was uploaded and used to train the AI feature and assign probability scores to each reference to predict relevance. A minimum threshold was established where the AI feature correctly identified all manually screened relevant references. The AI feature was tested on a separate set of references (n=72,686) from the May 2019 to April 2020 monthly searches. The testing set was used to determine an optimal threshold that ensured >99% of relevant references would continue to be added to Health Evidence. The performance of AI-assisted screening at the title and abstract screening level was evaluated using recall, specificity, precision, negative predictive value, and the number of references removed by AI. The number and percentage of references removed by AI-assisted screening and the change in monthly manual screening time were estimated using an implementation reference set (n=272,253) from November 2020 to 2023.

**Results:**

The minimum threshold in the training set of references was 0.068, which correctly removed 37% (n=16,122) of nonrelevant references. Analysis of the testing set identified an optimal threshold of 0.17, which removed 51,706 (71.14%) references using AI-assisted screening. A slight decrease in recall between the 0.068 minimum threshold (99.68%) and the 0.17 optimal threshold (94.84%) was noted, resulting in four missed references included via manual screening at the full-text level. This was accompanied by an increase in specificity from 35.95% to 71.70%, doubling the proportion of references AI-assisted screening correctly predicted as not relevant. Over 3 years of implementation, the number of references requiring manual screening was reduced by 70%, reducing the time spent manually screening by an estimated 382 hours.

**Conclusions:**

Given the magnitude of newly published peer-reviewed evidence, the curation of evidence supports decision makers in making informed decisions. AI-assisted screening can be an important tool to supplement manual screening and reduce the number of references that require manual screening, ensuring that the continued availability of curated high-quality synthesis evidence in public health is possible.

## Introduction

### Background

Public health programs, services, and policies aim to promote health and prevent injury and disease across populations [1]. Public health initiatives cover a breadth of topics, including water and air quality testing, infectious disease surveillance, vaccine provision, alcohol and tobacco sales legislation, and school nutrition programs. Public health practitioners and policy makers are expected to seek the best available evidence to inform decisions on implementing effective interventions to improve the health and well-being of communities and populations. Evidence-informed decision-making in public health involves using the best available evidence from research, local context, community or political preferences, and public health resources to improve health outcomes [2]. The use of rigorous evidence syntheses is a key component in an evidence-informed approach [3]. Synthesized evidence, such as systematic reviews, brings together findings from all studies on a specific research question to contribute to public health decision-making [4]. Variability in the methodological quality of evidence syntheses, time constraints, and resource limitations in using synthesized evidence are barriers to achieving evidence-informed public health decisions [5-7]. In 2005, Health Evidence was established to address these challenges by facilitating access to high-quality evidence syntheses, offering public health practitioners a one-stop shop for preappraised syntheses relevant to public health [8].

### Health Evidence

**Figure 1.**
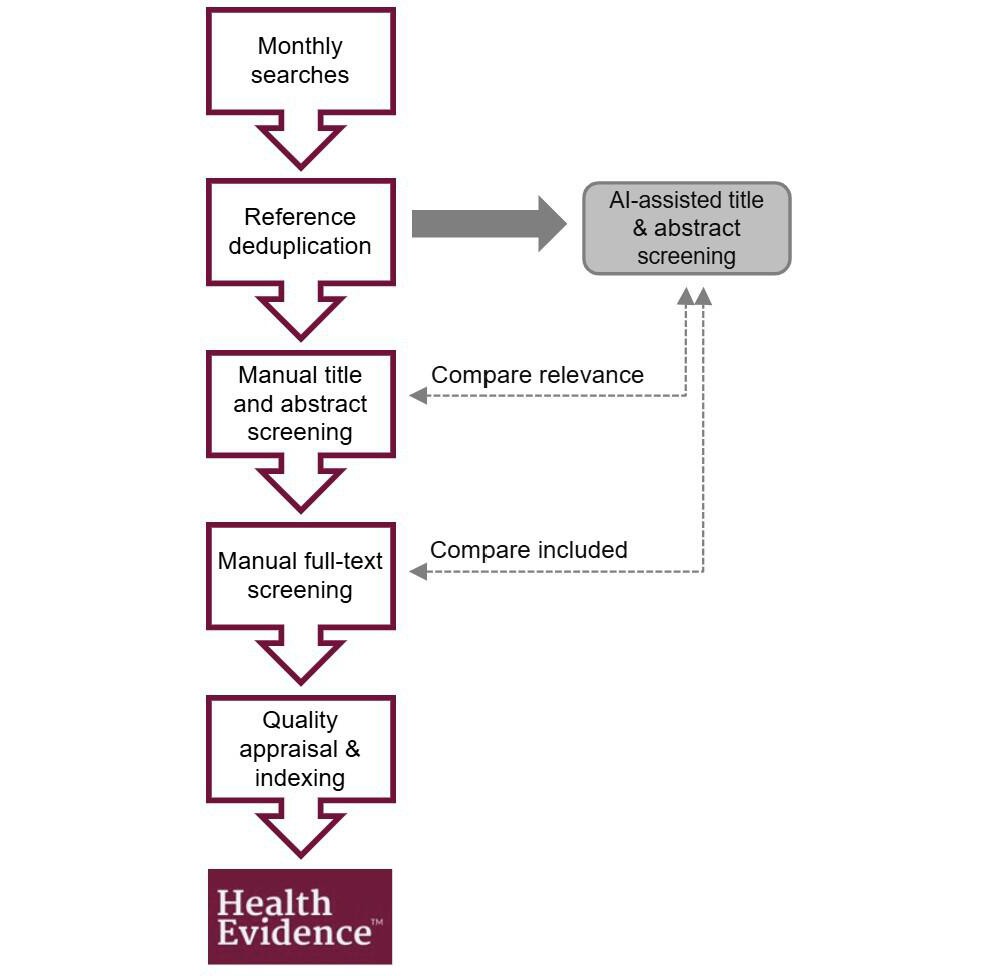
Health Evidence monthly workflow and AI-assisted screening integration and analysis. AI: artificial intelligence.

Health Evidence hosts over 10,000 quality appraisals of published evidence syntheses on the effectiveness and cost-effectiveness of public health and health promotion interventions [8]. Over the past 10 years, over half a million users have accessed Health Evidence worldwide. To keep Health Evidence up to date with the most recent evidence syntheses, monthly database searches of MEDLINE, Embase, CINAHL, and PsycINFO, monthly hand searches of Cochrane Library, Health Systems Evidence, and ACCESSSS Smart Search, and annual database searches of BIOSIS, SPORTDiscus, and Sociological Abstracts are conducted. Duplicates are removed, and the titles and abstracts of all search results are screened manually for relevance. The full texts of all potentially relevant syntheses are screened using 5 criteria for inclusion (Textbox 1) [9]. References that meet all 5 inclusion criteria are quality appraised by two independent raters using the Health Evidence Quality Assessment Tool, indexed with keywords, and added to the Health Evidence website [10]. An overview of the Health Evidence workflow is illustrated in Figure 1.

As public health spans a broad array of topics, the search strategy must also be broad to ensure all relevant public health reviews are captured. Previous developmental work at Health Evidence concluded that it is more efficient to search the published literature for systematic reviews and then screen for those relevant to public health, as opposed to developing a search strategy specific to all public health topics [11]. The last two decades have seen exponential growth in the number of published evidence syntheses [12-15], leading to a notable increase in the search results retrieved from monthly and annual database searches for Health Evidence. From 2018 to 2023, the average monthly database search results increased by 47%, from 8829 to 13,007. As the volume of published evidence syntheses grows, updating Health Evidence has become increasingly resource-intensive. Innovative screening methods using artificial intelligence (AI) are a potential solution to reduce the number of references requiring manual screening and ensure that Health Evidence remains feasible to maintain.

Textbox 1. **Health Evidence criteria.**

- Is this a review paper?
- Is the review relevant to public health or health promotion practice?
- Is the effectiveness of an intervention, program, service, or policy the subject of the review?
- Is evidence on outcomes included?
- Is the search strategy described?

### Application of AI to Maintain Health Evidence

AI commonly refers to the interdisciplinary study and development of models engineered to perform varied levels of automation that would typically require human intelligence [16,17]. Machine learning is a subset of AI that involves algorithms that autonomously learn patterns from the data they are trained on without being explicitly programmed [16]. Natural language processing is closely tied to machine learning algorithms, which involve computer systems’ ability to automatically process human language by segmenting unstructured text into smaller chunks and applying natural language processing techniques until a desirable pipeline is achieved [18]. The use of AI to semiautomate the screening of studies for relevance within the systematic review process has been described as promising [19-21].

In 2018, Health Evidence began investigating the use of AI to reduce the burden of manual title and abstract screening using an existing web-based review management platform, DistillerSR. This platform was selected based on the team’s familiarity with the software, its low cost, and the availability of live technical support [22]. The DistillerSR Preview & Rank feature is a logistic regression classifier that learns from manual screening decisions by applying a supervised machine learning model through a support vector machine nonprobabilistic binary linear classifier [23]. The Preview & Rank feature is preconfigured, internally tuned, and uses language-modeling-based feature representation to learn from the language patterns provided in the training data. To prepare the training data, standard preprocessing steps are applied, including tokenization, stop-word removal, and normalization. By learning from the data provided by labeled manual screening decisions, this feature assigns references a score between 0 and 1 to predict the probability that the reference is relevant, with scores closer to 1 more likely to be relevant. These assigned probability scores can be used to test the performance of the AI feature using multiple thresholds and select an optimal threshold by assessing the proportion of correctly predicted references compared to manual screening results. The optimal threshold is defined as the probability score that correctly identifies relevant references while correctly removing the greatest number of nonrelevant references. Reviewers can then remove references with a score below the optimal threshold without manual review. To address known challenges in using AI to support reference screening, quality assurance testing, and continuous monitoring is recommended to ensure that the AI feature continues to function as expected. These challenges may include variability in the content of the monthly searches and AI concept drift, for example, when novel phenomena emerge that were not captured in the training set [24]. The objectives of this project are to (1) assess the ability of AI-assisted screening to correctly predict nonrelevant references at the title and abstract level using an optimal threshold, and investigate the consistency of this performance over time; and (2) evaluate the impact of AI-assisted screening on the overall monthly manual screening set.

## Methods

### Ethical Considerations

As this is a quality improvement project that does not include participants or participant data, an ethics review was not required per the guidelines of the Hamilton Integrated Research Ethics Board [25].

### Datasets

Four types of reference sets from various database searches were used in this project: the “AI training set,” the “AI testing set,” the “quality assurance sets,” and the “implementation set.” The following sections describe these datasets in detail.

### Objective 1: Assessing the Ability of AI-Assisted Screening to Predict Nonrelevant References and Investigating Consistency in Performance Over Time

#### Phase 1: Training the AI Feature

The “AI training set” (n=43,273) included all evidence syntheses included in Health Evidence from 2005 to 2018 (n=6742) and a set of references that were deemed not relevant through manual title and abstract screening from January to August 2018 (n=36,531). The size of the “AI training set” was limited by the processing capacity of DistillerSR at the time; thus, only a subset of nonrelevant references (from 2018) was included. The “AI training set” was uploaded to DistillerSR, and the data were used to train the AI feature and assign a probability score to each reference in the set. The range of probability scores assigned to references was examined to establish a minimum threshold by identifying the probability score at the title and abstract level, where all references were correctly identified as relevant. The proportion of references identified as not relevant by AI-assisted screening at the minimum threshold was recorded.

#### Phase 2: Testing the AI Feature

The “AI testing set” included the manually screened search results from May 2019 to April 2020 (n=72,686). The “AI testing set” was uploaded to DistillerSR, and the trained AI feature described above was applied to assign probability scores to each reference prior to manual screening. Manual screening was conducted independently from the platform to ensure there was no indication of the manual screening result. This new set of references was used to test the performance of the AI-assisted screening feature by comparing (1) the results of the AI-assisted screening to the results of the manual title and abstract screening and (2) the number of missed references identified incorrectly as not relevant through AI-assisted screening to the references included at manual full-text screening and added to Health Evidence. Integration and analysis points in the Health Evidence workflow for comparing AI-assisted screening to manual screening are depicted in Figure 1.

A series of AI-screening tests were performed to identify the optimal threshold, defined as the threshold resulting in the highest number of references correctly predicted by AI as not relevant at the title and abstract level (ie, the maximum reduction in references to manually screen) with minimal references incorrectly excluded at the full-text screening level. References with a probability score below the optimal threshold can be removed without review, whereas references with a score equal to or greater than the optimal threshold require manual screening.

To examine the overall impact of these tested thresholds on title and abstract screening, performance was assessed by identifying true positives, true negatives, false positives, and false negatives. A true positive was a reference identified as relevant by AI-assisted and manual screening. A true negative was a reference identified as not relevant by AI-assisted and manual screening. False positive references were those identified by AI as relevant but assessed as not relevant in manual screening. False negative references were those identified by AI as not relevant but assessed as relevant in manual screening [26]. These values were used to calculate recall (the proportion of references correctly identified by AI as relevant out of all manually relevant references, also referred to as sensitivity), specificity (the proportion of references correctly identified by AI as not relevant out of all manually not relevant references), precision (the proportion of references correctly identified by AI as relevant out of all references predicted as relevant), and negative predictive value (the proportion of references correctly identified by AI as not relevant out of all references predicted as not relevant). The absolute number of references identified as not relevant by AI-assisted screening at each tested threshold was used to estimate the reduction in the number of references that require manual screening.

As the goal of the project was to use AI-assisted screening to reduce the number of references requiring manual screening, while minimizing false negatives after full text review, the negative predictive value was prioritized over other values, such as *F*_1_-score (which helps to identify a balance between precision and recall). A pragmatic approach was adopted to determine the optimal threshold to ensure that over 99% of all references added to Health Evidence through manual screening would have been captured if AI-assisted title and abstract screening had been integrated. Over 5 years (2015‐2019), the average number of references added to Health Evidence annually was 511, establishing an acceptable error rate of no more than five incorrectly excluded references (less than 1%) at the full-text screening level. The number of false negatives at both the title and abstract level and after manual full-text screening was calculated to determine the impact on the references that would ultimately be included in Health Evidence. Possible threshold scores were tested using the smallest probability score increments available on the platform (0.01), starting at the minimum threshold identified in Phase 1 up to five incorrectly excluded references. Incorrectly excluded references were examined to try to understand and explain patterns in what was ultimately added to Health Evidence at full-text screening but predicted as not relevant by AI-assisted title and abstract screening, for example, new phenomena that were not included in the “AI training set” (ie, COVID-19).

AI-assisted title and abstract screening using the optimal threshold established in the testing phase was then integrated into the Health Evidence monthly workflow. Implementation involved adapting the monthly workflow to upload the search results to the DistillerSR platform and apply the AI feature. The AI feature assigned probability scores to each uploaded reference, and the results were exported to EndNote (Clarivate). References with a probability score lower than the optimal threshold were removed from the screening set and not manually screened. Title and abstract, and full-text manual screening were conducted on the remaining references.

#### Phase 3: Performing Quality Assurance Checks of the AI Feature

Following the implementation of AI-assisted screening in the Health Evidence monthly workflow, a series of quality assurance checks were completed to ensure that the AI feature continued to perform as expected over time and identify when the AI model may need to be retrained. Quality assurance sets included references from the full 2020 annual database searches (n=9759) and monthly searches in July 2022 (n=4160), September 2023 (n=4482), and October 2024 (n=5201). Each set was manually screened and compared to AI-assisted screening results using the optimal threshold identified. The absolute number of false negatives was recorded.

### Objective 2: Evaluating the Impact of AI-Assisted Screening on Monthly Manual Screening

The “implementation set” (n=272,253) generated upon integrating AI-assisted screening into the Health Evidence monthly workflow was used to estimate the real-time impact of AI-assisted screening on monthly staff manual screening time. These included monthly search results from November 2020 to 2023. The number and percentage of references removed using the optimal threshold were recorded. A manual screening rate of 500 references per hour, based on 15 years of Health Evidence manual screening experience, was used to estimate the reduction in the number of hours of manual screening using AI-assisted screening to remove references below the optimal threshold.

## Results

### Objective 1: Assessing the Ability of AI-Assisted Screening to Predict Nonrelevant References and Investigating Consistency in Performance Over Time

#### Phase 1: Findings From Training the AI Feature

Using the “AI training set,” the minimum threshold was identified as 0.068. At 0.068, the AI feature correctly identified all relevant references and 37% (n=16,122) of the nonrelevant references at the title and abstract screening level.

#### Phase 2: Findings From Testing the AI Feature

Starting with the minimum threshold of 0.068, incremental threshold increases of 0.01 were assessed from 0.07 until five references incorrectly excluded at full-text manual screening were identified. Investigation of the five incorrectly excluded references found that three of these references were new phenomena (COVID-19) that did not exist in the “AI training set.” As such, those references were not considered AI-assisted screening errors, and incremental testing continued. To capture these types of new phenomena references, a separate hand search was implemented. Thresholds were tested, ranging from 0.07 to 0.18, at which time five references were incorrectly excluded and not explained by novel phenomena. Based on these findings, 0.17 was adopted as the optimal threshold. The overall performance (recall, specificity, precision, negative predictive value, and number removed by AI) of AI-assisted screening at the title and abstract screening level, and the number of false negative references at each screening level are reported in Table 1.

**Table 1.** Performance of AI^a^-assisted screening and number of false negatives in the “AI testing set.”

Threshold	Recall, %	Specificity, %	Precision, %	Negative predictive value, %	Removed by AI, n	False negatives, n		
						Title and abstract	Full text	Full text^b^
0.068	99.68	35.95	1.3	99.99	25,909	2	1	0
0.07	99.52	37.07	1.3	99.99	26,717	3	1	0
0.08	99.52	42.25	1.5	99.99	30,452	3	1	0
0.09	99.19	46.98	1.6	99.99	33,859	5	2	1
0.1	98.87	51.13	1.7	99.98	36,857	7	2	1
0.11	98.39	55.11	1.9	99.97	39,724	10	3	1
0.12	97.74	58.62	2	99.97	42,258	14	3	1
0.13	96.94	61.78	2.1	99.96	44,540	19	3	1
0.14	96.45	64.53	2.3	99.95	46,528	22	3	1
0.15	95.97	67.20	2.5	99.95	48,455	25	4	2
0.16	95.48	69.61	2.6	99.94	50,196	28	4	2
0.17	94.84	71.70	2.8	99.94	51,706	32	7	4
0.18	94.35	73.64	3	99.93	53,108	35	9	6

a AI: artificial intelligence.

b Adjusted number of false negative references at the full-text screening level, not considering new phenomena (ie, COVID-19).

A slight decrease in recall between the 0.068 minimum threshold (99.68%) and the 0.17 optimal threshold (94.84%) was noted, resulting in four missed references that were included at the full-text screening level using manual screening. This was accompanied by a substantial increase in specificity from 35.95% to 71.70%, doubling the proportion of references that AI-assisted screening correctly identified as not relevant and significantly reducing the number of search results requiring manual title and abstract screening by 51,706 (71.14%) references. These changes are reflected in the high negative predictive value, which remained above 99.9% at all thresholds tested. Precision was low, ranging from 1.3% to 3%, due to the high number of false positives that would subsequently undergo manual screening. This supports the need to continue conducting manual screening on the references identified as relevant by AI-assisted screening.

In a review of the four false negative references that were included through manual screening at the full-text level, it was determined that their exclusion related to types of intervention and population studied. For example, two focused on patients infected with HIV [27,28] and two on patients with type 2 diabetes mellitus [29,30]. Although technically meeting the broad Health Evidence topic criteria, their focus on secondary prevention in “patient” populations did not raise any concerns with their exclusion, as this is not generally a common focus area of local public health.

Starting in August 2020, the AI-assisted screening process using the 0.17 optimal threshold was integrated into the Health Evidence monthly workflow. Search results were uploaded to the DistillerSR platform monthly, and the AI feature was applied. References that scored below the optimal threshold were removed, and only the remaining references were screened manually.

#### Phase 3: Findings From Quality Assurance Checks of the AI Feature

The quality assurance check on the 2020 annual database search results (n=9759) found that AI-assisted screening removed 6830 (70%) of references, with no references incorrectly excluded. In the subsets of the monthly searches for July 2022 (n=4160), September 2023 (n=4482), and October 2024 (n=5201), only one reference from the July 2022 monthly searches was incorrectly excluded by the AI-assisted screening and included at the full-text screening level. Similar to the testing phase, this missed reference examined secondary prevention in patients with type 2 diabetes mellitus. In general, the quality assurance sets showed consistency in the overall percentage of references removed, ranging from 69% to 70%. This indicates that the model’s performance was maintained over a 3-year implementation period.

### Objective 2: Evaluating the Impact of AI-Assisted Screening on Monthly Manual Screening

Over 3 full years of implementation in the Health Evidence monthly workflow, AI-assisted screening eliminated 70% (n=190,966) of the references identified in our database searches, resulting in 81,287 references that underwent manual screening. On average, the use of AI-assisted screening decreased the annual number of references requiring manual title and abstract screening from 90,751 to 27,096.

Implementing AI-assisted screening in the Health Evidence monthly workflow has considerably impacted the required staff time to screen references for Health Evidence. It reduced the time spent manually screening by an estimated 382 hours over a 3-year period. Table 2 provides a specific breakdown of hours of screening time reduced per year.

**Table 2.** Comparison of estimates of annual time needed to screen reference sets.

	Year 1 (hours)	Year 2 (hours)	Year 3 (hours)
Estimated screening time without AI^a^-assisted screening	185.0	181.8	177.8
Estimated screening time with AI-assisted screening	54.5	54.7	53.4
Estimated time saved per year	130.5	127.1	124.4
Estimated cumulative time saved	130.5	257.6	381.9

a AI: artificial intelligence.

## Discussion

### Principal Findings

This project demonstrates that AI-assisted screening is an efficient strategy to be used alongside manual title and abstract screening, removing as many as 70% of nonrelevant references while incorrectly excluding fewer than 1% of references annually. Given the very small percentage of studies incorrectly excluded and their focus on secondary prevention in specific “patient” populations, we consider the “cost” of missed references to have minimal impact on public health decision-making and worth the benefits of reduced references to screen for our evidence platform. This risk-benefit analysis will likely differ for other curated evidence platforms. To increase access to high-quality and timely evidence, curated evidence platforms on specific topic areas continue to grow in popularity. Examples include Epistemonikos [31], Social Systems Evidence [32], the Joanna Briggs Institute Evidence-Based Practice Database [33], and Essential Evidence Plus [34]. The exponential growth of peer-reviewed published evidence is a key challenge for maintaining these types of platforms. Using an already created AI feature reduced some of the upfront costs that would usually be involved in developing a new AI model. The successful implementation of AI-assisted screening to support the Health Evidence monthly workflow provides an example of a process from which others could learn in maintaining large databases of curated evidence using an existing AI tool.

### Comparison to Prior Work

To our knowledge, this is the first study to evaluate the suitability of a preexisting AI-assisted screening tool to maintain a curated evidence platform; however, two systematic reviews and one scoping review on different but related topics provide some comparative insights [21,35,36]. Most applicable to this project’s context, a 2021 systematic review included 10 papers assessing machine learning approaches to identify high-quality, clinically relevant evidence in the biomedical literature. Recall ranged from 9% to 98% but was generally above 85%, specificity ranged from 76% to 88%, and precision ranged from 9% to 86% [35]. Looking at the use of AI in the conduct of systematic reviews, a 2015 systematic review included 44 papers assessing text mining approaches for study identification and reported a 30% to 70% workload savings with 95% recall [21]. Finally, a more recent 2022 scoping review of 47 papers assessed the role of automation throughout the systematic review process. Of the 28 papers that evaluated AI approaches for relevance screening, sensitivity or recall ranged from 75% to 100%, specificity from 19% to 99%, precision from 8% to 83%, median potential screening time saved ranged from 9 to 185 hours per review, with overall workload reduction ranging from 6.89% to 70.74%, and error rates between 0% and 22% [36]. Direct comparison of this project to the results of the studies identified in these reviews is challenging due to variations in the training, testing, and analysis approaches, as well as the inconsistent reporting of results. However, overall, the findings of these reviews align with those of this project, particularly concerning recall, specificity, and the reduction in the number of references that need to be screened manually.

### Lessons Learned

This project highlights several lessons learned on the use of preexisting AI tools for reference screening that may be important considerations for database management, specifically, or systematic review processes more generally. While the model training completed in this project was purpose-built for Health Evidence and cannot be directly applied to other platforms, describing the methods and results from this project may inform processes for others who are looking to reduce the number of manual references that need to be screened while minimizing false negatives. First, even with predeveloped software, the training, testing, and analysis of AI-assisted screening requires significant human resources. For the overall cost-benefit ratio to be favorable, the volume of references that require relevance screening should be substantial to justify the upfront investment. Curated evidence platforms like Health Evidence, which span a broad range of topics and generate over 10,000 search results each month, can find longer-term benefits in reduced screening time that justify the initial investment in training and testing. Second, access to large datasets that can be used as a reference standard for training and testing, such as what was available with Health Evidence as a longstanding curated evidence platform, is important to enable confidence in the adequate functioning of an AI-assisted approach for relevance screening. This approach may not be suited to smaller evidence platforms or systematic reviews that do not have access to such large datasets and reference standards. However, this approach may be transferable to systematic reviews with large search results, living systematic reviews, or systematic review updates if there is continuity in relevance criteria over time.

Third, concept drift is an important consideration for any AI model trained using historical data. Unlike AI models developed using continuous learning models, the model’s performance may degrade over time. Conducting quality assurance testing at predetermined intervals is recommended to evaluate performance over time. Over 4 years of quality assurance checks, one reference was found to be incorrectly excluded at the full-text screening level, demonstrating continued performance of the AI model and suggesting that model retraining is not yet required. Through ongoing annual quality assurance testing, we continue to monitor model performance. Through this work, we learned that if a new topic is identified, its impact on model performance should be investigated and compared to a pre-established threshold (eg, <1%). A separate hand search strategy can be implemented for distinct novel phenomena (eg, COVID-19). Continued monitoring for subtle shifts, such as new terminology, will inform if full retraining is required. A comprehensive assessment of optimal frequency and type of quality assurance practices is outside the scope of this project and is highly contextually dependent based on the purpose and scope of the platform, as well as how the sector evolves. These are important considerations, as frequent retraining of a model would require additional resources. While this project provides insight into the feasibility of integrating AI-assisted screening in similar contexts, the resources, training data, and monitoring practices required may vary depending on the complexity and scope of the research question or purpose of the curated platform.

Finally, while successfully applied to reduce the overall number of references requiring manual title and abstract screening, the results of this project do not support the use of AI screening as a replacement for manual screening. A thoughtful and cautious approach to establish where integration of AI could be most useful for similar types of platforms or projects is warranted.

### Implications for Practice

A key advantage of using AI-assisted screening to support Health Evidence is reducing the staff time required for title and abstract screening. While the full costs of training and implementing the AI feature were not calculated, the reduced screening time permits highly trained staff to be reallocated to more complex tasks in the Health Evidence monthly workflow that cannot be reliably augmented by AI. This ensures that public health decision makers can access quality appraised, newly emerging systematic reviews even more quickly. An advantage of using this AI-assisted screening approach is that it does not require frequent retraining for relevance screening. In contrast, training of new staff is needed when using only manual screening for reasons such as staff turnover or deployment to other project priorities. These factors help to reduce the resources required to keep Health Evidence up to date, increasing its long-term sustainability.

### Future Directions

The use of AI in database management and evidence synthesis is a rapidly evolving area with many gaps and areas for future research. To our knowledge, there are no other published reports on the practical application and real-world use of pre-existing AI tools to support curated evidence platforms. Studies published to date have explored a wide variety of AI approaches using different tools, for different purposes, and using different evaluation metrics, thus limiting the ability to draw overarching recommendations for AI use in practice, specifically for curated platforms. The authors encourage others to report on these types of continuous quality improvement projects to share learnings on the practical application of AI.

In this project, we used a pre-existing AI tool with the goal to minimize the number of references that need to be manually screened. For this purpose, the most relevant metrics to assess model performance included recall, specificity, negative predictive value, and absolute number of false negatives. In other studies of AI performance, *F*_1_-score and the area under the receiver-operating characteristic curve are used when a balance between recall and specificity is required. Due to the second screening at the full text level, we did not choose to focus on minimizing false positives at this stage. As AI tools continue to evolve, future work could explore a greater focus on this, as well as including *F*_1_-scores and area under the receiver-operating characteristic curve, to further general understanding of model functionality and optimization. Future work could also explore the use of explainable AI to better understand results and integrate learnings to improve the AI model. Investigating and comparing the value and risks of generative AI models compared to deterministic AI models for AI-assisted screening, including the use of continuous or incremental learning approaches, rather than manual thresholds, as used in this project, may offer further reductions in manual screening time. Finally, exploring additional applications of AI for other aspects of the evidence synthesis or Health Evidence monthly workflow, such as indexing, data extraction, and quality appraisal, and considering key issues regarding the ethical and equitable use of AI in database management and evidence synthesis are areas requiring further investigation.

### Conclusions

Given the magnitude of newly published peer-reviewed evidence, the curation of evidence supports decision makers in making informed decisions. AI-assisted screening can be an important tool to supplement manual screening and reduce the number of references that require manual screening, ensuring that the continued availability of curated high-quality synthesis evidence in public health is possible.

## Abbreviations

AI: artificial intelligence
